# A nomogram diagnostic cardiovascular disease in patients with erythrodermic psoriasis in Chinese

**DOI:** 10.3389/fimmu.2023.1159957

**Published:** 2023-06-02

**Authors:** Yue-Min Zou, Dong-Mei Zhou, Man-Ning Wu, Xin-Yuan Zhao

**Affiliations:** ^1^ Beijing University of Chinese Medicine, Beijing, China; ^2^ Beijing Hospital of Traditional Chinese Medicine, Capital Medical University, Beijing, China

**Keywords:** nomogram, erythrodermic psoriasis, cardiovascular disease, diagnostic, psoriasis

## Abstract

**Objective:**

Patients with erythrodermic psoriasis (EP) are associated with an increased risk of cardiovascular disease (CVD), because of the more severe inflammation in the skin areas. This study aimed to develop a diagnostic model for the risk of CVD in EP patients based on the available features and multidimensional clinical data.

**Methods:**

A total of 298 EP patients from Beijing Hospital of Traditional Chinese Medicine were retrospectively included in this study from May 5^th^, 2008, to March 3^rd^, 2022. Of them, 213 patients were selected as the development set by random sampling, and clinical parameters were analyzed by univariate and backward stepwise regression. Whereas the remaining 85 patients were randomly selected as the validation set. The model performance was later assessed in terms of discrimination, calibration, and clinical usefulness.

**Results:**

In the development set, the CVD rate was 9%, which was independently correlated with age, glycated albumin (GA>17%), smoking, albumin (ALB<40 g/L), and lipoprotein(a) (Lp(a)>300 mg/L). The area under the ROC curve (AUC) value was 0.83 (95% confidence interval CI, 0.73,0.93). For the validation set of EP patients, the AUC value was 0.85 (95%CI, 0.76,0.94). According to decision curve analysis, our model exhibited favorable clinical applicability

**Conclusion:**

EP patients with age, GA>17%, smoking, ALB<40 g/L, and Lp(a)>300 mg/L are associated with a higher risk of CVD. The nomogram model performs well in predicting the probability of CVD in EP patients, which may help improve perioperative strategies and good treatment outcomes.

## 1 Introduction

Erythroderma psoriasis (EP) is a disease that causes skin desquamation and widespread erythema on over 80–90% of the body surface area (BSA) (1). It is linked with a wide range of additional systemic problems, including electrolyte imbalance, dehydration, infection, edema, cachexia, and cardiovascular disease (CVD). Recently, psoriasis is suggested to be associated with various cardiovascular risk factors, and it has become a common cause of cardiovascular mortality in patients with severe psoriasis (2). There are unified underlying mechanisms of inflammatory pathways, cellular mediators, and genetic susceptibility between psoriasis and CVD (3). Body surface evaporation in erythrodermic patients leads to massive fluid loss, probably due to the significantly increased blood flow in the skin, and patients are prone to tachycardia, which may induce the occurrence of CVDs such as high-output heart failure, a fatal threat to EP patients (4). The risk of cardiovascular event in psoriasis patients increases by 1.5 times compared with the ordinary people (5). CVD is a major public health concern, and artery-related diseases have caused more than one-third of total mortality (6). Therefore, it is necessary to develop a model for predicting the risk of concomitant cardiovascular illness in individuals with EP, since severe psoriasis will raise the risk of CVD by 25% compared with those with mild psoriasis (7, 8). As confirmed by an increasing number of studies, EP is closely related to CVD. Patients with EP have concurrent CVD, which adds to the difficulty in treatment and increases the risk of mortality.

Furthermore, CVDs are mostly diagnosed based on coronary angiography, coronary multilayer CT angiography, echocardiography, and other tests. These examinations are expensive, and patients who visit the dermatology department usually do not complete these tests, especially for young patients who are not routinely screened, which results in missed diagnosis and misdiagnosis of CVD, delays patient treatment and possibly increases the risk of death.

The risk factors for EP remain unclear, besides, there is no available tool to assess probability of CVD in patients with EP at present.

This study aimed to explore the risk factors for CVD in EP patients by conducting a large cross-sectional study on EP patients. Moreover, a nomogram model was constructed based on the variables collected from this cross-sectional study, which better predicted the risk of CVD in patients with EP.

## 2 Methods

### 2.1 General information

The patients were assigned randomly in a 7:3 ratio to two cohorts, namely the derivation cohort and the validation cohort. According to the principle of having a minimum of 10 outcome events per variable, the sample size of the training set has been deemed sufficient. In this study, altogether 298 patients with EP were retrospectively included from the Beijing Hospital of Traditional Chinese Medicine. Among them, 213 EP patients were assigned into the development cohort, while the remaining 85 into the validation cohort.

Before testing, clinical data of patients were gathered and examined, including demographic data (gender, age, current smoking, current alcohol consumption); comorbidities (diabetes, hypertension, hyperlipidemia, nonalcoholic liver disease (NAFLD), psoriatic arthritis, and anemia); laboratory indexes including glycated albumin (GA), serum uric acid (UA), alanine aminotransferase (ALT), aspartate aminotransferase (AST), total serum cholesterol (CHO), total serum triglycerides (TG), calcium (Ca), potassium (K), albumin (Alb), C-reactive protein (CRP), homocysteine (HCY), serum creatinine (Cr), low-density lipoprotein cholesterol (LDL-C), high-density lipoprotein cholesterol (HDL-C), lipoprotein a (Lp(a)), amino-terminal pro-brain natriuretic peptide (NT-proBNP), and other risk factors that might affect cardiovascular prognosis, according to the clinical experience and research review.

The inclusion criteria for this study comprised patients who had previously been diagnosed with psoriasis that affected a minimum of 75-90% of the body surface area (BSA) and demonstrated prominent erythema and flaking.The CVDs involved in this study included coronary atherosclerotic heart disease such as myocardial ischemia and myocardial infarction, together with cerebrovascular disease like cerebral infarction.

### 2.2 Ethical review

The integrated electronic health record (EHR) system formulated by the Hospital Authority was used for clinical management. Data from the EHR system of Hospital Authority were de-identified and anonymized. This study was approved by Beijing Hospital of Traditional Chinese Medicine.

### 2.3 Statistical analysis

STATA 15.0 and SPSS 26.0 for Windows (IBM) were employed for statistical analysis. The reported statistical significance was two-sided, and P<0.05 was accepted as statistically significant. In this study, the patient test data were transformed into categorical variables by combining clinical significance and reported in a numerical form. Moreover, chi-square test and continuous correction test were conducted to compare categorical variables. Total column line plots were developed by a multivariate logistic regression model based on the test set. For variable selection, univariate logistic regression analysis was carried out, and variables with p-values <0.1 were retained as candidates. Thereafter, the candidate variables were incorporated into the multivariate logistic analysis. Regressions including the input method, forward stepwise regression, and backward stepwise regression were used. Finally, the model with the lowest Akaike information criterion (AIC) value was selected as the prediction model.

Columnar plots were evaluated by discrimination and calibration of the test and validation sets. The area under the curve (AUC) values were calculated for the test and validation sets, with the AUC value of 0.5 being undifferentiated and that of 1 being fully differentiated. Calibration plots were drawn to assess the calibration of the reformulated model by Hosmer-Lemeshow tests. Decision curve analysis (DCA) was performed to determine the clinical utility of the line plot by quantifying the net benefits at different threshold probabilities.

## 3 Results

### 3.1 Demographic and clinical features

A total of 305 patients aged over 18 years with a diagnosis of EP were included in this study, among them, 7 with missing data were excluded. At last, 298 patients were eventually included into this study, including 27 experiencing CVD. These clinical data were assigned by using the computer-generated Radom numbers into a development set (consisting of clinical data of 213 patients) and a validation set (including clinical data of 85 patients). The CVD did not change during the study period. First of all, balanced comparisons of demographic and study factors were conducted between the training and validation sets, which demonstrated that the two datasets were from the same population and comparable (**Table 1**).

**Table 1:** Demographic and clinical features of the participants.

Feature	Development set	Validation set	P
Age, n (%)			
<40	50(23.5)	17(20.0)	0.517
≥40	163(76.5)	68(80.0)	
Gender,n(%)			
Male	154(72.3)	68(80.0)	0.169
Female	59(27.7)	17(20.0)	
Hypertension,n(%)			
Yes	61(28.6)	24(28.2)	0.945
No	152(71.4)	61(71.8)	
Diabetes,n(%)			
Yes	27(12.7)	6(7.1)	0.163
No	186(87.3)	79(92.9)	
Anemia,n(%)			
Yes	28(13.1)	6(7.1)	0.136
No	185(86.9)	79(92.9)	
smoking,n(%)			
Yes	97(45.5)	41(48.2)	0.674
No	116(54.5)	44(51.8)	
drinking,n(%)			
Yes	102(47.9)	35(41.2)	0.294
No	111(52.1)	50(58.8)	
ALB,n(%)			
<40g/L	40(18.8)	19(22.3)	0.485
≥40g/L	173(81.2)	66(77.7)	
Abnormal liver function,n(%)			
Yes	26(12.2)	6(7.1)	0.195
No	187(87.8)	79(92.9)	
Cr,n(%)			
>84umol/L	12(5.6)	2(2.4)	0.365
>84umol/L	201(94.4)	83(97.6)	
Hypokalemia,n(%)			
Yes	22(10.3)	7(9.0)	0.582
NO	191(89.7)	78(91.0)	
splenomegaly,n(%)			
Yes	7(3.3)	1(1.2)	0.56
NO	206(96.7)	84(98.8)	
Hypocalcemia,n(%)			
Yes	138(85.9)	53(62.4)	0.692
No	75(14.1)	32(37.7)	
Psoriatic arthritis,n(%)			
Yes	35(16.4)	6(7.1)	0.034
NO	178(83.6)	79(92.9)	
Skin infections,n(%)			
Yes	26(12.2)	9(10.6)	0.719
NO	187(87.8)	75(89.4)	
Sinustachycardia,n(%)			
Yes	7(3.2)	5(5.9)	0.303
No	206(96.8)	80(94.1)	
Hyper			
homocysteinemia,n(%)			
Yes	44(20.7)	16(18.9)	0.722
No	169(79.3)	69(81.1)	
Respiratory tractin fections,n(%)			
Yes	35(16.4)	14(16.5)	0.994
No	178(83.6)	71(83.5)	
CRP,n(%)			
<1.8 mg/dL	99(46.5)	41(48.2)	0.784
≥1.8 mg/dL	114(53.5)	44(51.8)	
CHO,n(%)			
>5.2mmol/L	35(16.4)	17(20)	0.464
≤5.2mmol/L	178(83.6)	68(80)	
TG,n(%)			
>1.7mmol/L	84(39.4)	28(32.9)	0.296
≤1.7mmol/L	129(60.6)	57(67.1)	
LDL-C,n(%)			
>3.1mmol/L	36(16.9)	15(17.6)	0.877
≤3.1mmol/L	177(83.1)	70(82.4)	
HDL-C,n(%)			
>0.83mmol/L	77(36.2)	35(41.2)	0.419
≤0.83mmol/L	136(63.8)	50(58.8)	
UA,n(%)			
Female≥360umol/L	121(56.9)	50(58.8)	0.751
or male≥420umol/L			
Female<360umol/L	92(43.1)	35(41.2)	
or male<420umol/L			
Lp(a),n(%)			
>300mg/L	53(24.9)	26(30.6)	0.314
≤300mg/L	160(75.1)	59(69.4)	
ALT,n(%)			
<40 U/L	182(85.4)	74(87.1)	0.718
≥40 U/L	31(14.6)	11(12.9)	
AST,n(%)			
<35 U/L	175(82.2)	72(84.7)	0.598
≥35 U/L	38(17.8)	13(15.3)	
NAFLD,n(%)			
Yes	34(16.0)	9(10.6)	0.233
No	179(84.0)	76(89.4)	
GA,n(%)			
>17%	16(7.5)	3(3.5)	0.313
≤17%	197(92.5)	82(96.5)	
NTproBNP,n(%)			
≥125ng/L	23(10.8)	10(11.8)	0.126
<125mg/L	190(89.2)	75(88.2)	
cardiovascular			
disease,n(%)			
Yes	20(9.4)	7(8.2)	0.15
No	193(90.6)	78(91.8)	

Data are represented by n(%) unless otherwise stated. NAFLD, nonalcoholic liver disease; GA, glycated albumin; serum UA, serum uric acid; ALT, alanine aminotransferase; AST, aspartate aminotransferase; CHO, total serum cholesterol; TG, total serum triglycerides; Alb, albumin; CRP, C-reactive protein; Cr, serum creatinine; LDL-C, low-density lipoprotein cholesterol; HDL-C, high-density lipoprotein cholesterol; Lp(a), lipoprotein a; NT-proBNP, amino-terminal pro-brain natriuretic peptide.

**Figure 1:**
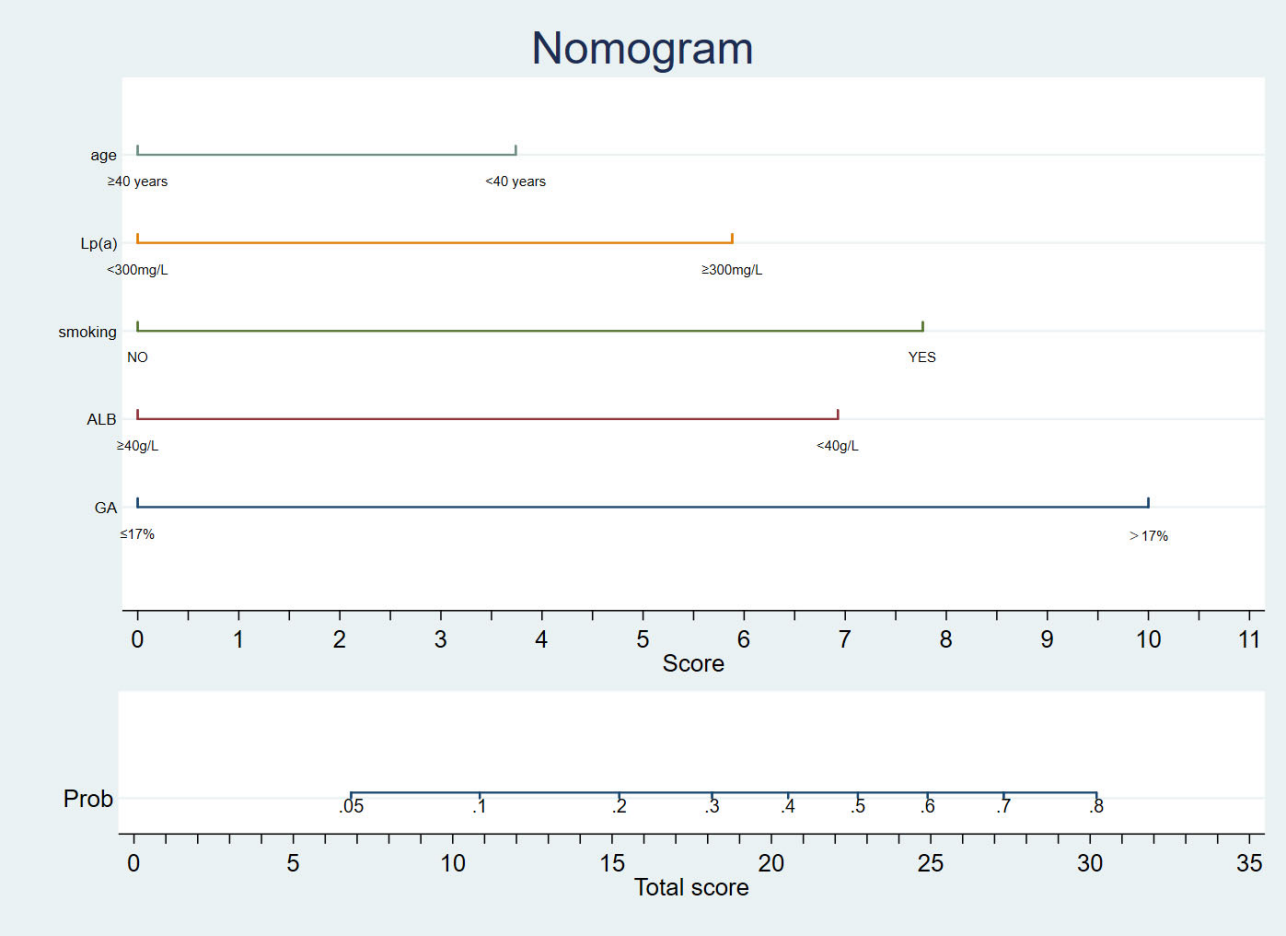
A nomogram diagnostic cardiovascular disease in patients with Erythrodermic psoriasis in Chinese.

Univariate analysis was later performed using a binary logistic regression model for all variables, as shown in **Table 2**. Thereafter, the 14 significant variables (p < 0.1) selected from univariate analysis were incorporated into multivariate logistic analysis. After a backward stepwise regression with the lowest AIC, four variables, namely, age, smoking, GA>17%, ALB<40 g/L, and Lp(a)>300 mg/L, were chosen for retention. Multivariate line graph of the probability of CVD in EP patients is displayed in **Figure 1**. As observed, each clinical characteristic corresponded to a specific point by plotting a straight line up to the point porridge. After summing the points located on the total point axis, the total posterior indicated the incidence of CVD by plotting the risk axis directly downward. For example, patients with the following characteristics: age of 40 years or older; Lp(a)>300 mg/L, smoking, Alb<40 g/L, and GA ≤ 17% interpreted a 37% probability of CVD among EP patients, and such results could be used for decision-making in treatment planning.

**Table 2:** Univariate logistic regression analysis of each indicator and diagnostic value in the development cohort.

Characteristic	Sig	ExpB	Lower	Upper
Gender	0.190	2.080	0.696	6.222
smoking	0.074	2.107	0.931	4.771
drinking	0.297	1.527	0.689	3.384
ALb	0.000	4.542	2.003	10.300
Abnormal liver	0.948	1.043	0.296	3.679
function				
Cr	0.114	2.955	0.771	11.321
Hypokalemia	0.800	1.178	0.332	4.180
splenomegaly	0.134	3.533	0.677	18.435
Hypocalcemia	0.127	2.079	0.812	5.324
Psoriatic arthritis	0.454	1.484	0.528	4.166
Skin infections	0.605	1.346	0.437	4.150
Sinustachycardia	0.359	2.088	0.433	10.063
Hyperhomocysteinemia	0.777	1.148	0.442	2.984
Hypertension	0.002	3.589	1.603	8.039
Respiratory	0.760	1.173	0.421	.262
tractin fections				
CRP	0.010	3.423	1.340	8.746
Anemia	0.017	3.163	1.226	8.163
Diabetes	0.061	2.582	0.959	6.953
CHO	0.495	1.398	0.534	3.654
TG	0.373	0.676	0.286	1.600
LDL-C	0.462	1.435	0.548	3.755
HDL-C	0.091	2.248	0.879	5.753
UA	0.030	2.823	1.105	7.216
Lp(a)	0.084	2.052	0.908	4.638
ALT	0.910	1.066	0.349	3.254
AST	0.462	1.435	0.548	3.755
NAFLD	0.023	2.839	1.155	6.977
CK	0.856	0.979	0.778	1.231
GA	0.000	7.554	2.678	21.313
NT-proBNP	0.000	5.146	2.085	12.698
age	0.015	0.339	0.142	0.810

NAFLD, nonalcoholic liver disease; GA, glycated albumin; serum UA, serum uric acid; ALT, alanine aminotransferase; AST, aspartate aminotransferase; CHO, total serum cholesterol; TG, total serum triglycerides; Alb, albumin; CRP, C-reactive protein; Cr, serum creatinine; LDL-C, low-density lipoprotein cholesterol; HDL-C, high-density lipoprotein cholesterol; Lp(a), lipoprotein a; NT-proBNP, amino-terminal pro-brain natriuretic peptide.

### 3.2 Development and evaluation of predictive column line graphs

**Figure 2:**
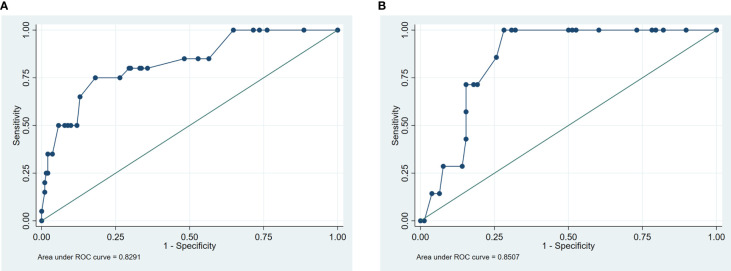
Receiver operating characteristic curve. **(A)** development set; **(B)** validation set.

**Figure 3:**
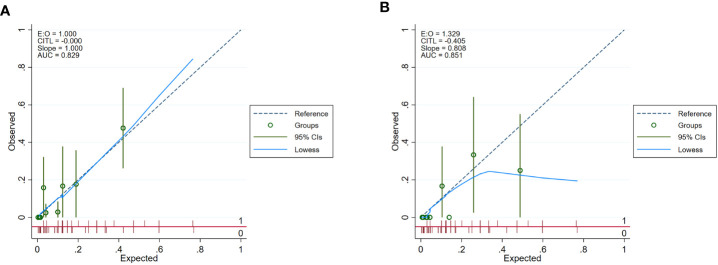
Calibration curves of the nomogram. **(A)** development set; **(B)** validation set.

**Figure 4:**
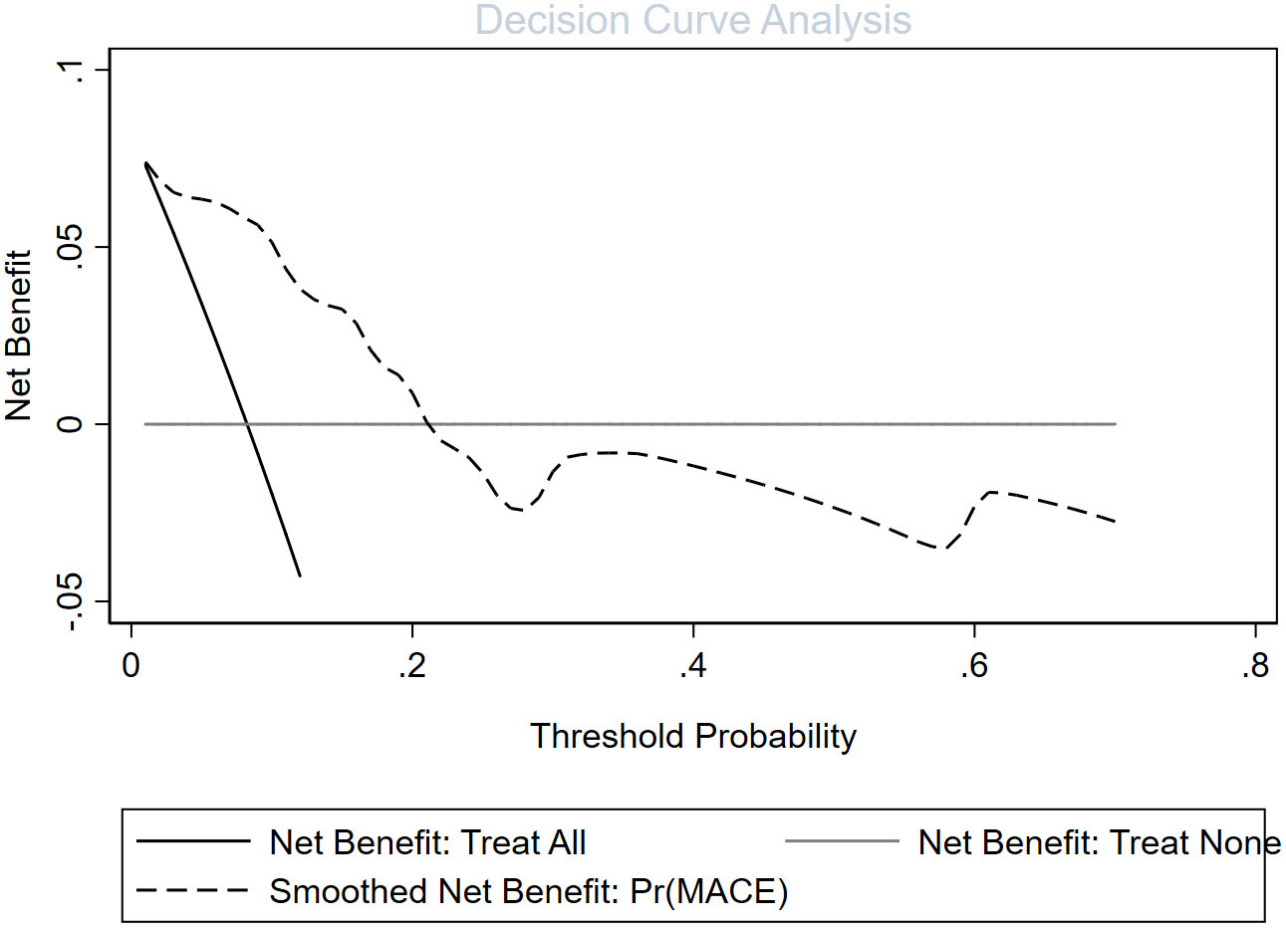
Evaluation of the decision curve analysis of a cardiovascular disease risk assessment model in patients with erythrodermic psoriasis.

In univariate and backward stepwise regression, each variable had a statistical significance level. The column line graphs achieved a good prediction performance with ROC values of 0.83 (95% confidence interval (CI) 0.73-0.93) and 0.85 (95% CI 0.76-0.94) for the development and validation sets, respectively (**Figure 2**). The calibrated E:O ratio in the validation set was 1.33 with a p-value of 0.85 (**Figure 3**). Besides, the Hosmer-Lemeshow (H-L) chi-square statistic was 4.51 with a p-value of 0.87, indicating the good model calibration. Furthermore, the clinical decision curve showed that the use of this column line plot predicted more benefits for patients with EP in terms of CVD incidence than the treat-all or no-treatment patient regimen if the patient had a threshold probability of 10%-22% (**Figure 4**). In this range (10%-22%), the net benefit was comparable.

## 4 Discussion

This study developed a new prediction tool to predict the probability of CVD in patients with EP by analyzing clinical inpatients from the Department of Dermatology, Beijing Hospital of Traditional Chinese Medicine. Data including laboratory tests, general demographic data, and basic vital sign data were extracted to help clinicians intervene psoriatic co-morbidities early and improve the quality of patient survival. DCA results indicated a positive net clinical benefit for patients with CVD, and ROC curves also demonstrated good differentiation.

Most of the existing models, including ours, can be used to predict the risk of CVD, such as the Framingham, SCORE, and QRISK7 models, however, all of them aim at normal adults. Notably, the cardiovascular risk prediction tools developed in asymptomatic adults have limited applicability in MS patients and lack the assessment of CVD risk in patients with EP. In the present study, as revealed by multifactorial logistic regression analysis and nomogram construction, age, smoking history, low Alb, and high GA level were risk factors for combined CVD in EP. The predictive molecules obtained by multivariate regression in this study can be easily accessible with low measurement costs, and relevant tests can be performed in general hospitals to obtain results to predict the risk of CVD.

A causal role of lipoproteins in the development of CVD has been demonstrated in previous mechanistic observations and evidence from genetic studies. The present study also suggested a strong correlation between them (9). Hypoproteinemia is associated with nutritional deterioration and disease-related inflammatory response (10). As reported in previous studies, hypoproteinemia is linked with an increased risk of CVD, regardless of whether the patient develops CVD or not (11). Moreover, it has been previously suggested that, hypoproteinemia plays an important role in the prediction of future cardiovascular events (12). GA is an indicator for glycemic control in diabetes mellitus (DM), a multifactorial disease associated with the incidence of CVD. GA has been incorporated in several CVD prediction models, but glycated hemoglobin was found to have a stronger correlation with CVD in this study (13). Anemia is frequently seen in patients with the acute coronary syndrome, heart failure, and coronary artery disease. Mild anemia may lead to an increased heart rate and affect the left ventricular diastolic function (10). This also indirectly confirms that anemia is a high-risk factor for the development of CVD (14, 15). Some studies have demonstrated that environmental factors such as smoking accelerate the development of CVD, and that nicotine in cigarettes increases the incidence of CVD by causing vasospasm and elevated blood pressure. In this study, a significant correlation was found between smoking and CVD in patients with EP (16). It has been shown that inflammation leads to the increased synthesis of apolipoprotein a. In the previous CVD prediction models, the risk of inflammation is underestimated, while in the current study, it was discovered that the increased apolipoprotein level resulted in an increased CVD risk in patients with EP (17). In this study, patients were divided according to age into > and < 40 years old, and logistic analysis revealed that patients aged <40 years had the most increased risk of CVD, which was consistent with previous studies (7).

Certain limitations should be noted in this study. First, Different therapies might change the cardiovascular risk in psoriasis patients, and the absence of information on psoriasis patient therapy in the included studies might have reduced the model validity (11). Second, our analyses on CVD were based on the present time point and psoriasis patients were not followed up for a long period, which might have caused some errors in the results. Third, older patients and those with more underlying disease were more likely to receive recommendations for hospitalization and were thus included in our study, which might be subject to selection bias. Fourth, some previously reported independent predictors of CVD, including cardiac ultrasound findings and electrocardiogram, were not included because of the insufficient data to avoid bias of too many missing values, therefore, the prognostic value of these factors for CVD was not estimated. Fifth, the Framingham, SCORE, and QRISK7 models were unavailable; thus, the nomo model was not compared with these two scoring models.

More external data are warranted to validate our model in future studies, so as to improve the result credibility and validity and improve the prediction model. More clinical information, such as treatment data, needs to be incorporated in the future to achieve a more multidimensional analysis and improve the model generalizability.

## 5 Conclusion

This study has developed and internally validated a new column line graph for predicting the risk of CVD in patients with EP. The ease of use and high accuracy of the line graph will help clinicians to personalize the prediction of CVD risk for each patient and improve the treatment advice for patients with EP.

## Data availability statement

The raw data supporting the conclusions of this article will be made available by the authors, without undue reservation.

## Ethics statement

Written informed consent was not obtained from the individual(s) for the publication of any potentially identifiable images or data included in this article.

## Author contributions

Y-MZ designed this work and revised this manuscript. D-MZ, M-NW and X-YZ prepared the data. Y-MZ integrated and analyzed the data. Y-MZ and D-MZ wrote this manuscript. All authors contributed to the article and approved the submitted version.
